# Novel perspectives on MSLN-targeted cancer therapy: from molecular mechanisms to clinical translation

**DOI:** 10.1080/15384047.2025.2603105

**Published:** 2025-12-16

**Authors:** Zhendong Wu, Xuefei Fu, Yuan Feng, Rong Zeng, Huan Qin, Kai Yao

**Affiliations:** a Institute of Visual Neuroscience and Stem Cell Engineering, Wuhan University of Science and Technology, Wuhan, China; b College of Life Sciences and Health, Wuhan University of Science and Technology, Wuhan, China

**Keywords:** Mesothelin, targeted therapy, immune escape, immunotherapy, cellular therapy

## Abstract

Mesothelin (MSLN) is a glycosylphosphatidylinositol (GPI)-anchored membrane protein that promotes malignant behaviors including tumor cell proliferation, migration and immune evasion through activation of multiple signaling pathways, such as MAPK/ERK and PI3K/AKT. MSLN is widely overexpressed in malignant tumors but shows low expression levels in normal tissues. This differential expression pattern renders MSLN an important clinical therapeutic target. Currently, MSLN-based tumor-targeting approaches predominantly involve antibody-drug conjugates (ADC), cancer vaccines, oncolytic viruses and chimeric antigen receptor T-cell (CAR-T) therapies. These therapeutic modalities have demonstrated encouraging efficacy in preclinical studies and phase I/II clinical trials. However, challenges such as unclear molecular mechanisms of MSLN signaling pathways and extracellular domain shedding impose limitations on targeted therapeutic strategies. Therefore, this review comprehensively discusses the gene and protein structures of MSLN, its biological functions, and related targeted therapeutic strategies, providing new insights into MSLN-targeted cancer therapy.

## Graphical abstract

**Figure:**
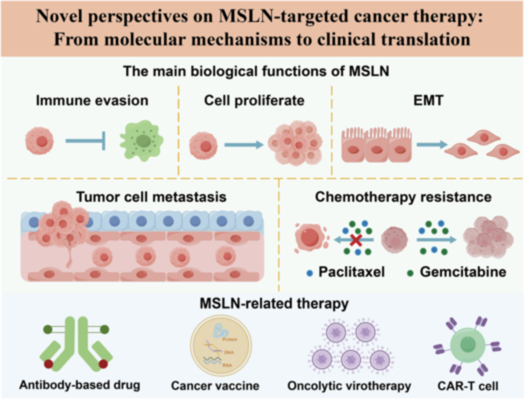

## 1 Introduction

Mesothelin (MSLN) is a glycosylphosphatidylinositol (GPI)-anchored cell surface glycoprotein that was first identified in 1992 by Willingham and colleagues.^1^^,^^2^ Clinical studies have demonstrated that MSLN is significantly overexpressed in various tumor tissues, while in healthy tissues, it is primarily found on the surface of mesothelial cells, with minimal expression in critical organs such as the lungs, kidneys, and liver.^3^ Furthermore, the aberrant expression of MSLN in tumors is closely associated with malignant phenotypes, including tumor cell proliferation, invasion, and resistance to chemotherapy, thereby positioning it as a potential tumor-specific therapeutic target.^4^

Currently, various mesothelin-targeted therapeutic strategies, including anti-mesothelin antibodies, antibody-drug conjugates (ADC), and Chimeric Antigen Receptor T-cell Therapy (CAR-T), have demonstrated promising anti-tumor efficacy in preclinical studies and some early-stage clinical trials. Extensive research has validated the differential expression of MSLN across various tumors and elucidated its mechanisms of action, providing a theoretical foundation for the application of MSLN in the treatment of multiple cancers. However, several challenges remain in the application of these therapies. For instance, tumor heterogeneity may lead to individual differences in treatment responses, off-target toxicity may induce non-specific side effects, and MSLN shedding may compromise the efficacy of targeted therapy. These issues can affect the effectiveness and safety of the treatment. Therefore, optimizing existing targeted therapy strategies to enhance therapeutic efficacy and reduce side effects will be a key focus of future research.

This review comprehensively discusses the molecular characteristics of MSLN and its translational potential as a therapeutic target for tumors. By examining the gene structure, molecular chaperones, and the signal transduction networks associated with MSLN, we systematically analyzed its multiple biological functions in tumorigenesis and development. Furthermore, drawing on the latest research findings, we evaluated the mechanisms and clinical translation status of various MSLN-targeted therapeutic strategies, thereby providing theoretical support for precision therapy targeting MSLN.

## 2 Structure of MSLN

**Figure 1.**
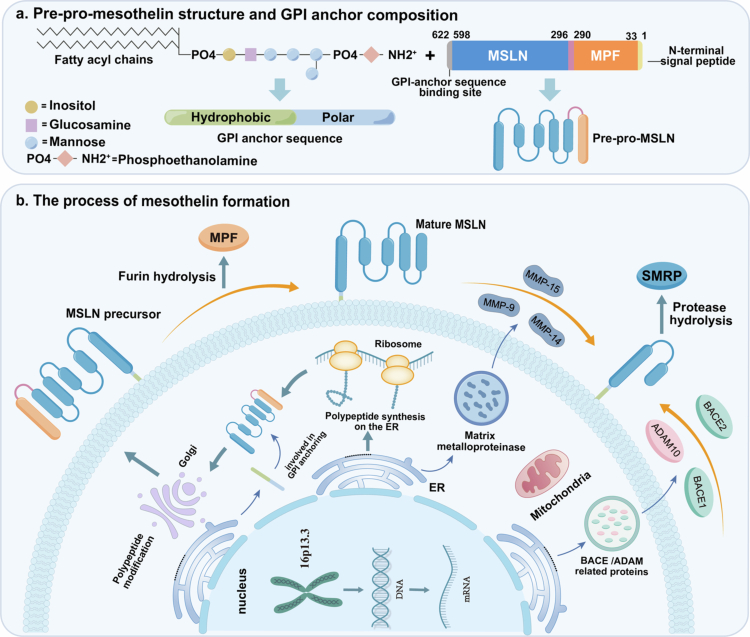
Structural characteristics of MSLN. (a) Structure of the GPI-anchored mesothelin precursor protein (Pre-pro-MSLN). The regions of pre-pro-mesothelin: signal peptide (yellow, 1–33), mature MPF (orange, 34–289), furin cleavage site (red, 290–296), mature mesothelin (MSLN, blue, 297–598) and GPI-attachment sequence (gray, 598–622). (b) The process of mesothelin formation. After transcription and translation, the precursor protein undergoes modifications in the endoplasmic reticulum (ER), associates with the GPI sequence and is ultimately localized to the cell membrane. The mesothelin precursor is cleaved by furin protease (or other proteases) to generate mature mesothelin and megakaryocyte potentiating factor (MPF). Mature mesothelin is anchored to the cell surface via the GPI anchor and can be further cleaved into soluble mesothelin-related peptides (SMRP) by other proteases, such as MMP9, BACE1 and ADAM17.

*MSLN* is located at the p13.3 locus of human chromosome 16. Its genomic structure consists of 15 exons and has a coding region length of 1884 bp, which enables the production of a precursor protein weighing approximately 69 kDa. The *N*-terminal signal peptide of this precursor protein directs it into the endoplasmic reticulum, where post-translational modifications are finalized. The C-terminus of the precursor protein features a typical GPI anchor signal sequence, comprising residues 599 to 622 (Figure 1A). This sequence is recognized and processed within the endoplasmic reticulum, thereby facilitating its binding to GPI-anchored proteins. Following its modification and transport by the Golgi apparatus, MSLN is ultimately localized at the cell membrane.^2^^,^^5^ The extracellular segment of the MSLN precursor protein is recognized and cleaved by furin protease at the R295 site, resulting in the production of two distinct fragments. One fragment is the mature MSLN, which has a molecular weight of approximately 41 kDa, while the other is the megakaryocyte-potentiating factor (MPF), with a molecular weight of approximately 31 kDa. Similar to most GPI-anchored proteins, mature MSLN can be hydrolyzed by various extracellular proteases, ultimately producing soluble mesothelin-related peptides (SMRP) (Figure 1B). The specific cleavage by different proteases can lead to the formation of various truncated proteins on the membrane. Furthermore, serum levels of SMRP have been shown to correlate with the clinical diagnosis and prognosis of various malignant tumors.^6^^,^^7^

An in-depth analysis of the protein structure of MSLN is crucial for understanding its biological functions. The full-length MSLN structure comprises five ARM repeat units and two HEAT repeat units, with each repeat unit consisting of two to three helices and a length of 27 to 37 amino acid residues. These repeat units are arranged in an alternating helical manner, forming a continuous hydrophobic core that ultimately folds into a highly ordered right-handed superhelical structure. This structural feature positions hydrophobic amino acids in the core to maintain conformational stability, while hydrophilic and charged amino acids are distributed on the periphery of the structure, facilitating specific interactions between proteins and between proteins and carbohydrates. The MSLN structure also contains two conserved disulfide bonds (C302/C326 and C442/C468), which contribute to its structural stability. Additionally, the C-terminal region of MSLN (M487-S598) is relatively loose, which may be associated with the dynamic regulation of its membrane localization.^8^^,^^9^

## 3 The molecular partner of MSLN - MUC16

The interaction between MSLN and MUC16 (CA125) plays a significant role in tumor progression. MUC16, a prominent member of the mucin family, is not only highly expressed in various tumors but also serves as a critical binding protein for MSLN.^10^ The binding of MSLN to MUC16 not only enhances the adhesion ability of tumor cells but also upregulates the expression of MMP7 through the PI3K/AKT signaling pathway, thereby strengthening the invasive capacity of these tumor cells.^11^ Clinical studies have demonstrated that the co-expression of MSLN and MUC16 is significantly correlated with tumor progression, adversely affecting patient prognosis.^12^

The extracellular segment of MSLN is divided into three distinct regions, with Region I serving as the primary site for MUC16 binding. Although Region I is a hotspot for antibody drug development, its competitive binding to natural ligands may influence the potency of antibody drugs, consequently reducing their therapeutic efficacy.^6^^,^^13^ To address this limitation, researchers are actively developing antibody drugs that target alternative regions. In vitro results have demonstrated the feasibility of this strategy. For instance, the HN1 antibody, which targets full-length MSLN, not only effectively inhibits the binding of MSLN to MUC16 but also induces cell death in MSLN-positive tumor cells through its antibody-dependent cellular cytotoxicity (ADCC) mechanism.^14^ Therefore, the development of therapeutic strategies targeting the interactions between MSLN and MUC16, along with the exploration of their combined application with existing therapies, is anticipated to yield new breakthroughs in tumor therapy.

## 4 The role of MSLN in cancer

Distant tumor metastasis is recognized as a significant contributor to poor prognosis in cancer patients. Accumulating evidence indicates that, in certain cancer subtypes, MSLN may facilitate distant metastasis through various molecular pathways. Analysis of clinical samples revealed a significant increase in MSLN expression levels in tumor tissues associated with brain metastasis, which correlates closely with a shortened survival period for patients. MSLN enhances the expression and phosphorylation of MET via the JNK signaling pathway, thereby increasing the ability of tumor cells to penetrate the blood-brain barrier.^15^ MSLN also promotes tumor invasion by upregulating matrix metalloproteinases, which facilitate this process by degrading the extracellular matrix. Specifically, MSLN activates the MAPK/ERK and JNK signaling pathways, inducing the expression of the transcription factor AP-1. The subsequent upregulation of MMP-7, driven by AP-1, enhances the metastatic and invasive capabilities of tumor cells.^16^ MSLN can also enhance the adhesion between tumor cells and mesothelial cells. This adhesion facilitates the binding of metastatic tumor cells to the mesothelial layer of peritoneal organs, ultimately leading to the establishment of metastatic foci.^17^ The SGK3/FOXO3 signaling pathway also plays a crucial role in tumor metastasis. Upon MUC16 binding to MSLN, this pathway is activated, leading to the downregulation of DKK1. The reduced levels of DKK1 diminish its inhibitory effect on the Wnt/β-catenin signaling pathway, thereby activating and expressing downstream target genes associated with tumor invasion.^18^ Blocking the binding of MSLN and MUC16 may be a promising approach to inhibiting tumor metastasis.

Epithelial-mesenchymal transition (EMT) is an early event in tumor metastasis, regulated by various molecules, including MSLN.^19^ Studies have demonstrated that MSLN overexpression leads to an increase in mesenchymal markers and a decrease in epithelial markers, thereby facilitating the EMT process.^20^ Conversely, the knockdown of MSLN expression reverses EMT, restores the expression of epithelial markers, and effectively inhibits tumor cell migration.^21^

MSLN induces resistance in tumor cells to chemotherapy. Specifically, MSLN inhibits paclitaxel-induced caspase activation through the PI3K/AKT signaling pathway, while simultaneously upregulating the expression of anti-apoptotic proteins such as Bcl-2 and Mcl-1, thereby establishing a dual resistance barrier.^22^ Similarly, in breast cancer models, MSLN also inhibits Bim-mediated apoptosis via the ERK signaling pathway, further enhancing the resistance of tumor cells to chemotherapy during metastasis.^23^ Additionally, clinical data analysis indicates that high expression levels of MSLN in stage IV colorectal cancer patients are negatively correlated with the chemotherapy response rate, underscoring the significant role of MSLN in chemoresistance.^24^

MSLN is closely associated with enhanced tumor cell proliferation. By activating the STAT3-cyclinE/CDK2 signaling axis, MSLN accelerates cell cycle progression, thereby promoting tumor cell proliferation.^25^ Additionally, MSLN increases the level of IL-6 secreted by tumor cells through the NF-κB signaling pathway.^26^ IL-6 not only promotes the expression of anti-apoptotic proteins such as Bcl-xL and Mcl-1, which help tumor cells resist TNFα-induced apoptosis, but also further drives cell cycle progression by promoting the phosphorylation of STAT3. This enables cancer cells to sustain growth and proliferation in the relatively harsh tumor microenvironment.^27^^,^^28^

**Figure 2.**
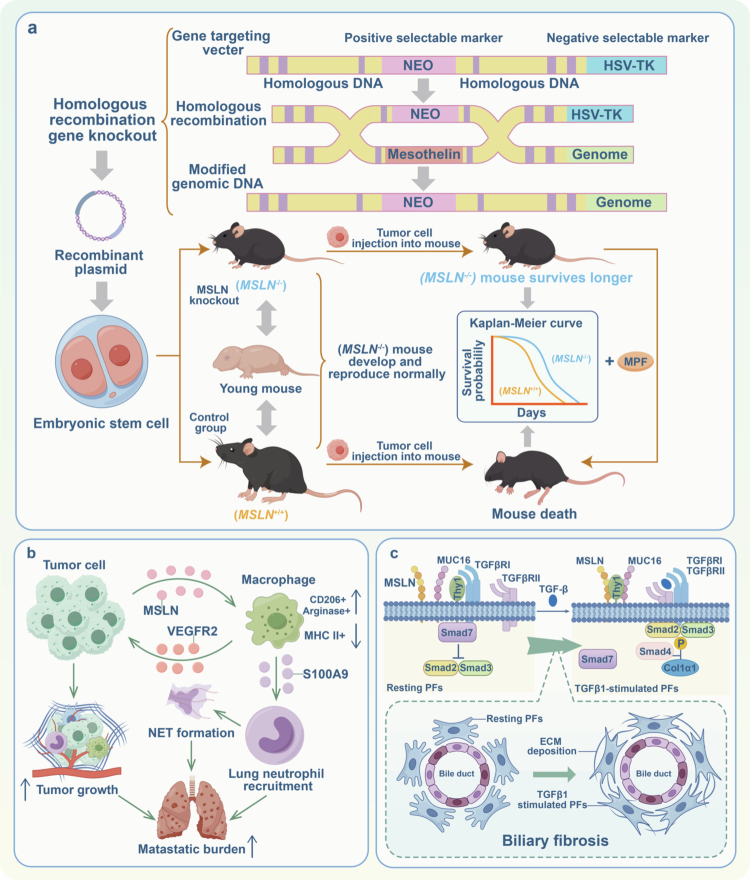
The function of MSLN in cancer. (a) MSLN and MPF promote tumor development. By knocking out the MSLN gene in mouse embryonic stem cells through homologous recombination, it was found that MSLN-deficient mice (*MSLN*^-/-^) could grow, develop and reproduce normally. However, compared to normal mice (*MSLN*^+/+^), *MSLN*^-/-^ mice showed a significantly prolonged survival after the transplantation of lung cancer cells. The Kaplan-Meier curve illustrates a difference in survival duration between the two groups of mice. Moreover, MPF supplementation in MSLN^-/-^ mice significantly shortened survival duration. (b) MSLN drives tumor metastasis via macrophage M2 polarization. Pancreatic cancer cells secrete MSLN which interacts with macrophages, promoting their polarization towards the M2 type, further enhancing the tumor burden and recruiting neutrophils to the lungs to facilitate metastasis. (c) MSLN promotes fibrosis progression. In wild-type portal fibroblasts, Thy-1 and MUC16 interact with TGFβRI receptors and SMAD7 inhibits the phosphorylation of SMAD2/3. Under TGFβ1 stimulation, the interaction between MSLN and Thy-1 increases, forming the TGFβRI/II-TGFβ complex, which leads to the phosphorylation of SMAD2/3/4, activating Col1a1 and promoting bile duct fibrosis.

*MSLN*-knockout mice exhibited no significant abnormalities in growth or reproduction, suggesting it is non-essential for normal mouse development.^29^ However, in a peritoneal metastasis model using A549 lung cancer cells, *MSLN*⁻/⁻ mice survived significantly longer than wild-type controls. This survival benefit was markedly diminished upon administration of the MSLN-derivative MPF. Notably, this difference was specific to the intraperitoneal model, as no significant disparity was observed in subcutaneous tumor growth. These findings imply that MPF promotes tumor progression specifically by modulating the intraperitoneal microenvironment (Figure 2A).^30^

Accumulating evidence indicates that MSLN orchestrates the phenotypes and functions of various immune cells within the tumor microenvironment, thereby fostering a complex immunosuppressive milieu. Elevated MSLN expression correlates strongly with reduced CD8+ T cell infiltration in the tumor microenvironment and poor patient prognosis. Mechanistic investigations have revealed that MSLN potentiates the activation of antigen-presenting cancer-associated fibroblasts (apCAFs). These activated apCAFs engage CD4+ T cells through MHC class II molecules, facilitating their conversion into immunosuppressive regulatory T cells, which in turn suppress the activation and functionality of CD8+ T cells.^31^ Moreover, MSLN can stimulate tumor-associated macrophages through paracrine mechanisms, leading to the secretion of significant amounts of cytokines such as VEGFA and S100A9. This process promotes tumor cell proliferation and the formation of neutrophil extracellular traps, thereby creating a favorable microenvironment for tumor metastasis (Figure 2B).^32^ MSLN also upregulates CD24 expression via the activation of the Wnt/β-catenin pathway, which induces pro-tumor M2 polarization of tumor-associated macrophages.^33^ Collectively, these studies illuminate the diverse mechanisms through which MSLN facilitates tumor immune escape, providing a crucial foundation for developing MSLN-targeted immunotherapy strategies.

MSLN has been implicated in the pathogenesis of liver fibrosis. Through in vitro studies, we found that MSLN inhibited the formation of thy-1-TGFβ1 complexes and regulated the activation of wild-type portal fibroblasts induced by TGFβ1 (Figure 2C). Furthermore, MSLN potentiates the proliferation of portal vein fibroblasts mediated by fibroblast growth factors. In gene knockout experiments, mice lacking MSLN exhibited markedly attenuated fibrosis, thereby corroborating the profibrotic role of MSLN.^34^ Notably, immunotherapy strategies targeting MSLN have been demonstrated to effectively alleviate liver fibrosis, suggesting that MSLN represents a promising therapeutic target for anti-fibrotic treatment.^35^

**Figure 3.**
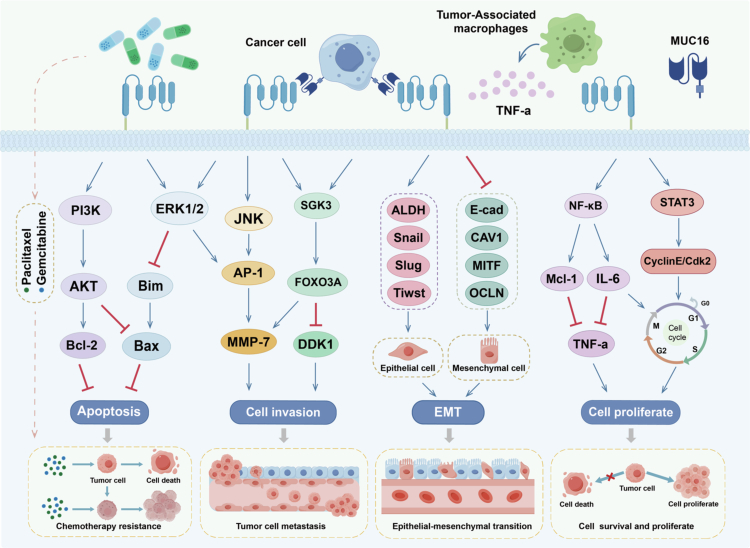
Signaling pathways associated with MSLN. Through interactions with MUC16, mesothelin can trigger multiple signaling pathways. The PI3K/Akt and ERK1/2 pathways enhance chemotherapy resistance by inhibiting pro-apoptotic proteins (such as Bim and Bax) while increasing anti-apoptotic proteins (such as Bcl-2). The JNK and SGK3/FOXO3 pathways promote tumor cell invasion and metastasis by upregulating the expression of MMP-7. Additionally, mesothelin further enhances tumor cell invasiveness by driving epithelial-mesenchymal transition (EMT). Overexpression of mesothelin also activates NF-κB and STAT3 signaling pathways. The downstream effects of NF-κB increase the expression of IL-6, which assists cancer cells in resisting apoptosis induced by TNF-*α* secreted from macrophages. Meanwhile, STAT3 signaling promotes the formation of the Cyclin E/CDK2 complex, thereby driving the cell cycle and increasing cell proliferation.

In summary, MSLN drives the progression of malignant tumors through multiple mechanisms, including regulating epithelial-mesenchymal transition, inducing chemotherapy resistance, and remodeling the tumor microenvironment (Figure 3). Additionally, its role in pathological processes such as liver fibrosis suggests broader functional implications. However, current understanding remains largely confined to cell line and animal models, and the regulatory networks of MSLN within the complex human tumor microenvironment, as well as its functional heterogeneity across different cancer types, require further clinical validation. Therefore, future research must prioritize large-scale clinical cohorts and integrate multi-omics data to precisely elucidate the biological functions of MSLN, thereby ultimately advancing the clinical success of MSLN-targeted therapies.

## 5 Expression differences of MSLN in tumors and clinical prognosis

Although the significant role of MSLN in tumor growth and metastasis has been widely recognized, whether MSLN can serve as a reliable tumor diagnostic marker or an independent prognostic indicator remains controversial. This controversy may stem from the complex and multifaceted biological functions of MSLN. In this section, we systematically synthesize the existing data on MSLN and its derivative SMRP as prognostic markers, elucidate their possible role in prognosis assessment, and provide valuable insights for future research and clinical applications (Table 1).

**Table 1.** MSLN expression in tumors.

Tumor type	Positive (%)	Test format	Test method	Prognosis	Reference
Ovarian cancer	5/10 (50%)	mRNA	SAGE	Inconsistent	[36]
	109/198 (55%)	m-MSLN	IHC		[36]
	34/48 (71%)	m-MSLN	IHC		[37]
	14/21 (67%)	SMRP	Elisa		[38]
Gastric cancer	124/212 (59%)	m-MSLN	IHC	Inconsistent	[39]
	476/958 (49%)	m-MSLN	IHC		[40]
	64/127 (50%)	m-MSLN	IHC		[41]
Pancreatic cancer	12/14 (86%)	m-MSLN	IHC	Worse	[42]
	22/24 (91%)	m-MSLN	IHC		[43]
	73/74 (98%)	SMRP	Elisa		[44]
	60/60 (100%)	m-MSLN	IHC		[45]
Acute myeloid leukemia	7/23 (35%)	m-MSLN	Flow cytometry	Worse	[46]
	598/1671 (36%)	mRNA	RNA-Seq		[47]
Epithelioid mesothelioma	97/115 (84%)	m-MSLN	IHC	Inconsistent	[48]
	39/39 (100%)	m-MSLN	IHC		[49]
	44/44 (100%)	m-MSLN	IHC		[50]
	177/356 (50%)	SMRP	Elisa		[51]
Sarcomatoid mesothelioma	2/10 (20%)	m-MSLN	IHC	Not applicable	[48]
	0/8 (0%)	m-MSLN	IHC		[50]
	0/10 (0%)	m-MSLN	IHC		[52]
Triple-negative breast cancer	30/71 (42%)	m-MSLN	IHC	Inconsistent	[53]
	29/43 (67%)	m-MSLN	IHC		[54]
	94/165 (57%)	m-MSLN	IHC		[55]
Cholangiocarcinoma	67/99 (68%)	m-MSLN	IHC	Worse	[56]
Endometrioid uterine adenocarcinoma	13/22 (59%)	m-MSLN	IHC	Worse	[57]
lung adenocarcinoma	78/148 (53%)	m-MSLN	IHC	Worse	[58]
	6/11 (55%)	m-MSLN	IHC		[59]

Abbreviations: SAGE: Serial analysis of gene expression; m-MSLN: membrane-bound mesothelin.

### 5.1 Ovarian cancer

Ovarian cancer is the gynecological malignancy with the highest mortality rate and a 5-year overall survival rate of approximately 47%. However, once distant metastasis occurs, patient survival rate plummets to 29%.^60^ MSLN was initially discovered in the OVCAR3 xenograft model and was identified as a specific antigen for ovarian cancer.^61^ Elucidating the relationship between the expression pattern of MSLN in ovarian cancer and clinical prognosis could help enhance its value in clinical practice.

By analyzing multiple SAGE (Serial Analysis of Gene Expression) databases containing both cancerous and normal tissues, investigators found that MSLN was specifically upregulated in approximately 50% of ovarian cancer cases, and this percentage was significantly higher than that of normal ovarian tissues.^36^ This was corroborated by a subsequent large-scale cohort study, in which MSLN was positively expressed in 70.8% of ovarian tumor samples.^37^ Additionally, MSLN can be shed from the cell surface into the peripheral circulation, suggesting its potential value as a liquid biopsy marker. This notion is supported by a study by Hassan et al, which demonstrated that MSLN concentrations were significantly elevated in the serum of 67% of ovarian cancer patients, thereby further substantiating the distinctive expression profile of MSLN expression in ovarian cancer.^38^

In recent years, MSLN has received widespread attention as a potential prognostic indicator for ovarian cancer. However, its prognostic significance remains contested. Some studies have shown that MSLN is associated with poor prognosis in patients with ovarian cancer. In high-grade serous ovarian cancer, elevated MSLN levels are typically associated with shorter progression-free survival (PFS) and overall survival (OS).^62^ Patients with high MSLN expression exhibit suboptimal responses to chemotherapy. In patients with advanced disease, MSLN is associated with poor survival outcomes, regardless of whether or not they have undergone tumor cytoreductive surgery.^63^ Conversely, other investigations did not find a significant association between MSLN expression and prognosis.^64^ Additionally, some studies have shown that diffuse expression of MSLN is associated with longer survival, though this association is only present in a small percentage of patients.^36^ Overall, although MSLN plays an important role in ovarian cancer, its utility as a prognostic marker remains unestablished and warrants further investigation to validate its prognostic value.

### 5.2 Gastric cancer

To investigate the expression pattern of MSLN in gastric cancer, Baba et al. analyzed tissue samples from 212 patients with gastric cancer. The results revealed that 59% of the patients exhibited positive MSLN expression. Notably, MSLN appears to be associated with a favorable prognosis in patients with gastric cancer. In patients with advanced stage disease, those with MSLN-positive tumors demonstrated longer survival times.^39^ Another study examining polymorphisms in the MSLN gene also demonstrated that certain mutations in the MSLN gene, such as rs3764247, were associated with a lower risk of gastric cancer and that patients harboring these mutations had significantly longer survival.^65^

However, accumulating evidence from other studies suggests that MSLN may portend a poor prognosis for patients with gastric cancer. Shin et al. demonstrated that MSLN not only exhibited elevated expression in 49.7% of cases, but was also significantly associated with reduced postoperative disease-free survival and an increased risk of peritoneal recurrence in patients.^40^ Furthermore, the expression pattern of MSLN is dynamic rather than static, demonstrating variability correlating with gastric cancer progression. Specifically, positive expression rates in stage I and IV tumors were 39% and 56%, respectively.^41^ These findings indicate that MSLN may play an important role in gastric cancer development, especially in tumor invasion depth and lymph node metastasis.^66^^,^^67^ Analogous to observations in ovarian cancer, the role of MSLN as a prognostic marker in gastric cancer remains incompletely elucidated, warranting more studies to verify its clinical utility in this malignancy.

### 5.3 Pancreatic cancer

Pancreatic ductal adenocarcinoma (PDAC) is characterized by challenges in early detection, pronounced heterogeneity and a propensity for distant metastasis.^68^ Accumulating evidence demonstrates that MSLN exhibits minimal expression in normal pancreatic tissues or adjacent non-malignant tissues, whereas it demonstrates robust expression in the majority of pancreatic cancer tissues, with this differential expression pattern being statistically significant.^42^^,^^43^ Moreover, serum soluble MSLN levels in pancreatic cancer patients are frequently elevated, indicating its potential utility as a biomarker for this malignancy.^44^

Concerning the impact of MSLN on the prognosis of patients with pancreatic cancer, the available evidence is relatively consistent. Pancreatic intraepithelial neoplasia (PanIN) represents an early neoplastic precursor lesion in pancreatic cancer development and is strongly associated with pancreatic cancer progression. MSLN is considered a significant driver in facilitating the progression of PanIN, with particularly elevated expression levels observed in high-grade PanIN (PanIN-3) and invasive adenocarcinoma.^45^ A tissue microarray survival analysis demonstrated that MSLN expression could predict the likelihood of short-term, cancer-related death after surgery in patients with pancreatic cancer. Its predictive power even surpassed that of traditional pathological features such as tumor size, lymph node metastasis, and histological grade.^69^ Moreover, the co-expression of MSLN and MUC16 was associated with higher histologic grade, vascular invasion and tumor recurrence rates in pancreatic cancer and portended poorer recurrence-free survival and overall survival in affected patients.^70^^,^^71^ Although the prognostic role of MSLN in patients with ovarian and gastric cancers remains controversial, data derived from pancreatic cancer studies consistently indicate that high MSLN expression portends an unfavorable prognosis for patients.

### 5.4 Acute myeloid leukemia

MSLN has been extensively explored as an immunotherapeutic target in clinical trials for a variety of solid tumors, yet its therapeutic potential in hematological tumors has not yet been fully elucidated, necessitating more in-depth studies to characterize the expression pattern of MSLN in hematologic malignancies. Emerging evidence demonstrates that in acute myeloid leukemia (AML) patients, MSLN is predominantly expressed in the CD45dim/SSC-Alow myeloid cell subpopulation and exhibits minimal expression in mature lymphocytes and monocytes.^46^ This unique expression pattern not only substantiates the marked cellular selectivity of MSLN in AML, but also suggests that MSLN may play a role in regulating specific biological processes within AML cells.

MSLN exhibits minimal expression in normal bone marrow, but demonstrates substantial expression in the myeloid cells of a subset of AML patients. As the disease achieves remission, MSLN expression progressively returns to baseline levels in patients.^72^ This phenomenon was further corroborated through transcriptome analysis by Meshinchi et al. (*n* = 2,051). This study additionally identified the presence of soluble MSLN in the serum of patients with AML, underscoring its potential as a liquid biopsy biomarker for this malignancy.^47^ The prognostic impact of MSLN is particularly pronounced in specific molecular subgroups of AML. In the core binding factor (CBF)-abnormal subtype, MSLN overexpression was observed in 72% of cases.^73^ Survival analysis revealed that among AML patients harboring CBF abnormalities, the risk of recurrence approximated 51% in MSLN-positive patients compared with 32% in MSLN-negative patients. The corresponding disease-free survival rates were 46% and 64%, respectively, indicating that low MSLN expression is associated with favorable clinical outcomes.^74^ Moreover, MSLN overexpression was significantly associated with an increased risk of developing extramedullary disease (EMD). The incidence of EMD in MSLN-positive patients was 27.8%, compared with 18.8% in MSLN-negative patients. This difference was markedly accentuated in patients with relapsed/refractory (R/R) AML. Although MSLN-positive cases comprised only 35% of R/R cases, they represented 53% of EMD cases, compared with 18.9% among MSLN-negative cases. These findings suggest that MSLN-positive patients may experience poorer survival outcomes.^47^

### 5.5 Mesothelioma

Mesothelioma is a rare malignant tumor characterized by highly aggressive behavior. Due to limited therapeutic options and rapid disease progression, the prognosis of mesothelioma patients is generally poor.^75^ Given the significant overexpression of MSLN in mesothelioma, this molecule has emerged as a focal point in mesothelioma research, and its soluble derivative SMRP has been validated as an important biomarker for both diagnosis and prognostic assessment of mesothelioma.^76^

Mesothelioma is histopathologically classified into three subtypes: epithelioid, sarcomatoid, and biphasic. MSLN exhibits distinct expression patterns across different mesothelioma subtypes.^77^ In epithelioid mesothelioma, MSLN demonstrates high expression levels and can serve as an ancillary diagnostic marker for this subtype; conversely, sarcomatoid mesothelioma typically displays relatively low MSLN expression.^48^^,^^49^ For biphasic mesothelioma, which contains both epithelioid and sarcomatoid components, MSLN expression exhibits regional heterogeneity: epithelioid regions generally maintain robust MSLN positivity, whereas sarcomatoid regions are predominantly negative or weakly positive.^50^ Consequently, the overall MSLN expression level in biphasic mesothelioma typically falls between that of epithelioid and sarcomatoid subtypes, with the specific expression pattern potentially determined by the proportion and distribution of these two components within the tumor.^78^

Within mesothelioma, high MSLN expression does not necessarily portend a worse prognosis. Related studies have demonstrated that in epithelioid malignant pleural mesothelioma (MPM), higher MSLN expression levels are significantly associated with longer median survival in patients.^52^ A potential explanation is that MSLN may regulate type I collagen in the tumor microenvironment to maintain its structural integrity, thereby limiting the invasion of malignant cells to some extent. However, this mechanism is currently based solely on correlative analyzes of clinical samples, and the specific causal relationships await further verification through functional experiments.^79^

When analyzing the impact of MSLN on patient prognosis, it is essential to comprehensively consider the expression levels of SMRP in serum. The SMRP positivity rate in patients with epithelioid mesothelioma is as high as 58%, and its expression levels show a strong correlation with tumor burden and disease stage.^51^ Furthermore, SMRP may serve as a predictive biomarker for treatment response to immune checkpoint inhibitors, with higher baseline levels potentially indicating shorter survival duration.^80^ A survival analysis based on serum SMRP levels revealed that patients with serum SMRP levels above 1 nM experienced significantly shortened survival. Another large-scale survival analysis encompassing 579 MPM patients also confirmed that elevated serum SMRP expression is associated with reduced overall survival.^81^^,^^82^ Therefore, serum SMRP levels can not only serve as an auxiliary diagnostic tool, but also predict disease progression and recurrence risk by reflecting dynamic changes in tumor burden.

### 5.6 Triple-negative breast cancer

Triple-negative breast cancer (TNBC) is a highly aggressive subtype of breast cancer, characterized by the absence of expression of the estrogen receptor (ER), progesterone receptor (PR), and human epidermal growth factor receptor 2 (HER2). Clinically, treatment options for patients with locally advanced or metastatic disease are relatively limited.^83^ In a retrospective study of breast cancer, 42.3% of TNBC patients were found to have positive MSLN expression.^53^ Similarly, Tchou's team reported a high expression rate of MSLN in TNBC, with a positivity rate as high as 67%.^54^ Thuwajit's team further explored the subtype specificity of MSLN expression in breast cancer. Their study showed that the expression rate of MSLN was highest in TNBC, at 57%, while it was significantly lower in HER2-positive and luminal types, at 14.9% and 1.9%, respectively, highlighting the potential of MSLN as a specific immunotherapeutic target for TNBC.^55^ It is worth noting that current studies have shown no significant association between MSLN expression and long-term survival outcomes, including overall survival, disease-specific survival, and disease-free survival.^84^ Therefore, the specific molecular mechanisms and regulatory pathways of MSLN in the development of TNBC still need to be further explored.

### 5.7 Other types of cancer

MSLN also has research value in other types of malignant tumors. For example, in cholangiocarcinoma (CCA), a retrospective analysis was conducted on 99 CCA tumor samples that had been surgically resected between 2000 and 2014. Researchers found that 68% of the tumor samples had positive MSLN expression.^56^ Interestingly, the level of MSLN expression was positively correlated with the distance of the primary tumor from the liver. The median density of positive cells increased from 42% in intrahepatic cholangiocarcinoma (ICC) to 60% in hilar extrahepatic cholangiocarcinoma (H-ECC) and then to 80% in distal extrahepatic cholangiocarcinoma (D-ECC). In addition, MSLN expression was significantly associated with a shorter overall survival in patients.^85^^,^^86^ This suggests its potential value as a prognostic biomarker for CCA.

MSLN exhibits heterogeneous expression in endometrial cancer, with varying positivity rates across different histological subtypes: the highest MSLN positivity rate is found in clear cell carcinoma (71.4%), followed by serous carcinoma (56.5%) and carcinosarcoma (50.0%); whereas in endometrioid carcinoma, the positivity rate ranges from 45.5% to 59%.^57^^,^^87^ The Miyamoto team also found that MSLN positivity in endometrial cancer is associated with deep myometrial invasion and lymphovascular invasion, and is confirmed as an independent prognostic indicator for progression-free survival and overall survival; especially when MSLN is co-expressed with CA125, it indicates a worse prognosis for patients.^88^

In addition, in the study of pulmonary adenocarcinoma, the Miettinen team reported that approximately 53% of pulmonary adenocarcinomas express MSLN.^58^Subsequent studies have further confirmed the widespread expression of MSLN in pulmonary adenocarcinoma and its association with KRAS mutations.^59^^,^^89^ More importantly, a large-sample study by the Kachala team showed that high MSLN expression is an independent predictor of the invasive phenotype of pulmonary adenocarcinoma, significantly associated with increased risk of recurrence and shortened overall survival.^89^ These findings indicate that MSLN is not only a potential prognostic biomarker for pulmonary adenocarcinoma but also a therapeutic target worthy of further exploration.

**Figure 4.**
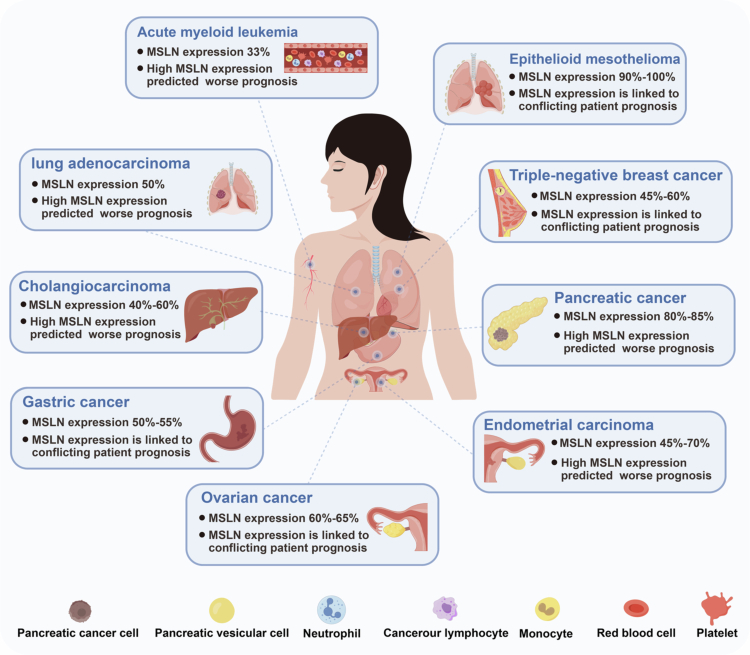
Prognostic impact and distribution pattern of MSLN in tumor. MSLN demonstrates variable expression levels across different malignancies, ranging from 33% in acute myeloid leukemia to 90%-100% in malignant mesothelioma. High expression of MSLN is associated with a poorer prognosis in tumors such as acute myeloid leukemia, lung adenocarcinoma, and pancreatic cancer; however, its prognostic value remains unclear in cancers including malignant mesothelioma, gastric cancer, ovarian cancer, and triple-negative breast cancer.

MSLN has been extensively studied across multiple tumor types, with its expression patterns and prognostic value varying according to tumor type (Figure 4). However, whether MSLN can serve as a marker of poor patient prognosis remains controversial. In MPM, higher MSLN expression levels are positively correlated with improved patient prognosis. Conversely, elevated serum SMRP levels predict shortened patient survival. Therefore, further studies are necessary to clarify the definition of “MSLN-positive” and to adopt more comprehensive methods for evaluating actual MSLN expression levels in tumor tissues. Furthermore, previous studies detecting MSLN expression have largely lacked simultaneous analysis of intratumoral cell subpopulations. Traditional immunohistochemical or RNA sequencing methods primarily reflect overall MSLN expression levels in tumor tissues and fail to distinguish whether expression originates from tumor cells themselves or other cell subpopulations within the tumor. This analytical gap may lead to misjudgment of MSLN's biological functions. Future research should integrate technologies such as multiplex immunofluorescence, flow cytometry, or single-cell RNA sequencing to accurately elucidate the cellular origin of MSLN expression while clarifying its relationship with the infiltration levels and spatial distribution of different immune cell subpopulations. This approach is crucial for accurately evaluating the prognostic value of MSLN, identifying populations that can truly benefit from MSLN-targeted therapy, and understanding the complete landscape of MSLN as an immunotherapeutic target.

## 6 MSLN-targeted therapy

Currently, MSLN-targeted therapeutic strategies primarily encompass antibody-based drugs, cancer vaccines, oncolytic viruses, and CAR-T cell immunotherapy. The safety and efficacy of these therapeutic approaches have been evaluated in multiple preclinical and clinical trials. Table 2 systematically summarizes the clinical trial data of the aforementioned therapeutic strategies, including the number of enrolled patients, trial phases, therapeutic advantages and limitations, and can provide important references for the design and optimization of more subsequent clinical trial protocols.

**Table 2.** Clinical development overview of MSLN-Targeted therapeutic agent.

Investigational agent	phase	Trial	Enrollment	Results	Clinical trial	Advantages	Potential limitations	References
^Amatuximab^	I	Amatuximab	17	18% SD, 82% PD	NCT01018784	It can directly kill tumor cells through ADCC; The treatment was well-tolerated.; It has significant potential for combination treatments.	Anti-drug antibodies impair therapeutic efficacy; Poor penetration ability in solid tumors and insufficient intratumoral concentration; Limited efficacy as a single agent.	[90]
	II	Amatuximab or Pemetrexed and Cisplatin	89	PFS: 6.1months OS: 14.8months	NCT00738582			[91]
	II	Amatuximab or Pemetrexed and Cisplatin	108	Higher chemotherapy shown be associated longer OS	NCT02357147			[92]
ABBV-428	I	ABBV-428	25	36% SD, 64% PD	NCT02955251	TME-dependent activation mechanism, which significantly reduces systemic toxicity.	Anti-drug antibodies have a relatively high incidence rate.	[93]
Immunotoxin SS1P	I	SSIP	34	12% MR, 56% SD, 29% PD	NCT00066651	Possesses dual effects of direct killing and immune activation; Stable efficacy is achieved with monotherapy; Combination therapy can significantly enhance therapeutic efficacy.	Strong immunogenicity limits multi-cycle treatment; The efficacy of single-agent therapy is limited; Prone to non-specific vascular toxicity.	[94]
	I	SSIP	24	4% PR, 50% SD, 46% PD	NCT00006981			[95]
Immunotoxin LMB-100	I	LMB-100	24	50%SD, 50%PD	NCT02317419 NCT02798536			[96]
	Ib/II	LMB-100 or Nab-paclitaxel	20	5%PR, 35%SD	NCT02810418			[97]
	II	LMB-100 or Pembrolizumab	21	PFS: 3.3mouths, OS: 11.9mouths	NCT03644550			[98]
Anetumab ravtansine (AR)	I	AR	148	8.1% ORR, 45% SD	NCT01439152	The safety profile is manageable, Reduced toxicity compared to traditional chemotherapy; It possesses a bystander effect, suitable for tumor heterogeneity; Capable of Enhancing Chemosensitivity.	Limited efficacy of monotherapy; Interference from soluble MSLN; Efficacy is strictly dependent on high MSLN expression in tumors.	[99]
	II	AR or Vinorelbine	248	The therapeutic effect of AR is not superior to vinorelbine	NCT02610140			[100]
	Ib	AR or Doxorubicin and Pegylated liposomal	65	27.7% ORR, PFS:5mouths MDOR:7.6mouths	NCT02751918			[101]
	I/II	AR or Pembrolizumab	48	11% ORR, 50%DCR PFS:12.2mouths	NCT03126630			[102]
BMS-986148	I/IIa	BMS-986148 or Nivolumab	126	20% PR, 85% DCR PFS:6.5mouths	NCT02341625			[103]
CRS-207	I	CRS-207	17	37% of patients survived for ≥ 15 months	NCT00585845	Combination therapy with synergistic efficacy enhancement; Remodel the TME and activate immunity.	Dependent on tumor antigen expression; Limited efficacy of monotherapy; Risk of specific adverse reactions.	[104]
	Ib	CRS-207 or pemetrexed/cisplatin	37	57%ORR,86%DCR, PFS:7.5months, OS:14.7months	NCT01675765			[105]
	II	GVAX Pancreas or CRS-207	93	13%DCR, OS:5.9months	NCT02004262			[106]
ONCOS-102	I	ONCOS-102	12	40%SD, OS:9.3months	NCT01598129	Activate tumor immunity and break immune tolerance; Exhibit systemic immune effects; Possess significant potential for combination therapy.	Limited efficacy of monotherapy; Restricted administration routes; Low infection efficiency.	[107]
MSLN-CAR-T	I	CART-meso	15	PFS:2.1months, 0%ORR	NCT02159716	Strong targeting specificity and low off-target risk; Antitumor activity can be enhanced through structural modification; Demonstrates therapeutic potential for refractory solid tumors; Has potential combined therapeutic value.	CAR-T cells have poor persistence and insufficient tumor infiltration; The tumor microenvironment exerts inhibitory effects on CAR-T cells; The antigen heterogeneity and the risk of drug resistance exist.	[108]
	I	KT032	8	33%ORR,66.7%SD,100%DCR PFS:5.5months, OS:10.5months	ChiCTR2100046544			[109]
	I	7 × 19 CAR-T cells	2	One patient achieved CR; One patient experienced PD	NCT03198546			[110]
	I	αPD-1-mesoCAR-T cell or apatinib	1	PFS:5months, OS:17months	NCT03615313			[111]

Abbreviations: SD: stable disease; PD: progressive disease; PFS: progression-free survival; OS: overall survival; PR: partial response; ORR: objective response rate; MDOR: median duration of response; DCR: disease control rate; ADCC: antibody-dependent cellular cytotoxicity; TME: tumor microenvironment.

### 6.1 Antibody-based drugs

**Figure 5.**
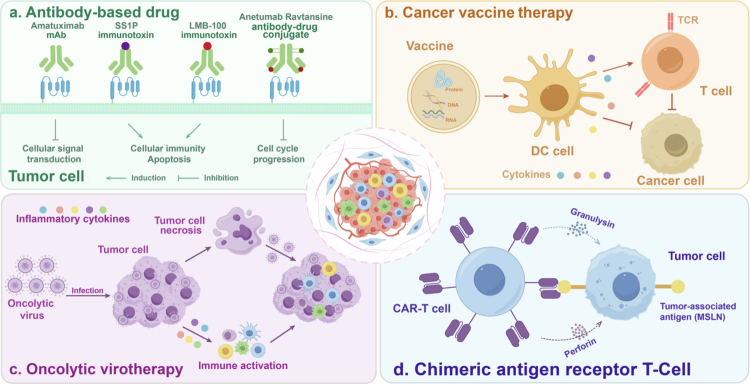
Targeted therapeutic strategies for MSLN. (a) Antibody-based drug: This approach uses monoclonal antibodies, immunotoxins and antibody-drug conjugates (ADC) to target MSLN on the surface of tumor cells, thereby killing the tumor cells. (b) Cancer vaccine therapy: Cancer vaccines stimulate dendritic cells to activate T cells, which target and eliminate cancer cells by recognizing tumor-associated antigens such as MSLN. (c) Oncolytic virotherapy: Oncolytic viruses infect and kill tumor cells, inducing tumor necrosis. At the same time, they activate immune responses, release inflammatory cytokines and promote immune cells to suppress the tumor. (d) Treatment of MSLN-expressing tumors with CAR-T cell therapy.

The development of antibody drugs targeting MSLN has been rapid, covering a variety of strategies such as monoclonal antibodies, immunotoxins and antibody-drug conjugates (Figure 5A). Several MSLN-based therapeutic strategies have shown preliminary therapeutic effects in clinical studies. This section will provide an overview of the current therapeutic landscape of MSLN-targeted antibody drugs, propose targeted improvement strategies, and lay a theoretical foundation for the development of next-generation antibody drugs.

#### 6.1.1 Monoclonal antibody

Amatuximab (MORAb-009) is a monoclonal antibody that specifically targets MSLN. In vitro experiments have shown that the antibody effectively inhibits cell adhesion and kills tumor cells through ADCC.^112^ In the completed Phase I clinical trials, amatuximab was administered to patients with MSLN-expressing cancers, including mesothelioma, pancreatic cancer, and ovarian cancer. No severe dose-limiting toxicity was observed in the subjects at the maximum tolerated dose (MTD), indicating that amatuximab has favorable safety and tolerability profiles.^113^ In another clinical study, patients with mesothelioma, pancreatic cancer, or other mesothelin-positive solid tumors received amatuximab monotherapy for four weeks. The treatment regimen consisted of weekly intravenous injections with escalating doses ranging from 50 mg/m² to 200 mg/m². Post-treatment efficacy assessments showed that only 17% of patients achieved stable disease (SD).^90^ This demonstrates the relatively limited clinical efficacy of amatuximab monotherapy.

To enhance the clinical efficacy of amatuximab, combination therapy strategies have become a key focus of research. In a phase II clinical trial, 89 patients with advanced pleural mesothelioma received a treatment regimen of amatuximab in combination with pemetrexed and cisplatin. Efficacy assessments showed that 33 patients achieved PR and 42 patients achieved SD, resulting in a 90% disease control rate. Combination therapy significantly prolonged the median survival time compared with amatuximab monotherapy. No severe adverse events were observed during treatment, indicating that the combination regimen was well tolerated by patients.^91^ In another study involving patients with unresectable MPM who received amatuximab combined with pemetrexed and cisplatin, the association between amatuximab exposure level and treatment response was evaluated. Results demonstrated a positive correlation between amatuximab exposure level and patients' OS, further confirming the clinical utility of this combination therapy regimen.^92^

Monoclonal antibodies activate immune cells to attack tumors by binding to specific antigens on the surface of tumor cells. However, the efficacy of such antibodies is often limited owing to the complex immunosuppressive state in the tumor microenvironment (such as high expression of PD-L1).^114^ In contrast, bispecific antibodies that can simultaneously target MSLN and immune co-stimulatory molecules can effectively address the issue of insufficient immune activation associated with monoclonal antibody therapy in the tumor microenvironment. Bispecific antibody drugs targeting MSLN are gradually entering the clinical research stage. ABBV-428 is a novel bispecific antibody that achieves tumor-specific immune activation by specifically binding to MSLN on the surface of tumor cells and to CD40 on antigen-presenting cells. In a Phase I clinical trial involving patients with advanced ovarian cancer or mesothelioma, 25 patients evaluable for efficacy were treated with 3.6 mg/kg (intravenous infusion every 2 weeks). Efficacy assessment showed that 9 patients (36%) achieved SD; moreover, no significant dose-limiting toxicities were observed during treatment, indicating that the regimen was well-tolerated. Further analysis also revealed that MSLN expression levels were positively correlated with prolonged PFS in patients. This suggests that MSLN can serve as a predictive biomarker of treatment response to this antibody.^93^ In subsequent clinical development, the therapeutic efficacy of ABBV-428 may be enhanced through optimizing the treatment regimen and refining patient selection criteria, thus providing better treatment options for patients with MSLN-positive tumors.

#### 6.1.2 Immunotoxin

Immunotoxins are primarily constructed by fusing antibody domains that target specific antigens with cytotoxins through genetic recombinant techniques. These immunotoxins recognize specific antigens on the surface of target cells via single-chain antibody fragments (scFv), thereby delivering the toxin to the target cells and inducing apoptosis.^115^ SS1P is a recombinant immunotoxin consisting of a high-affinity anti-MSLN scFv fused to a Pseudomonas aeruginosa exotoxin (PE).^116^ SS1P can enter tumor cells via endocytosis, whereupon the toxin is released into the cytoplasm to induce apoptosis by inhibiting protein synthesis.^117^ In the Phase I clinical trial for mesothelioma, ovarian cancer, and pancreatic cancer, no significant cardiotoxicity of SS1P was observed in patients, with its common adverse effects mainly including pleurisy and elevated serum creatinine levels.^94^ However, in vitro experiments showed that even at an SS1P concentration of 2000 ng/mL, the agent failed to achieve 50% or greater cytotoxicity in MSLN-positive AML cells, suggesting that its therapeutic efficacy for AML is relatively limited.^118^ Subsequent studies also revealed that SS1P exhibits strong immunogenicity. Notably, 75% of the patients developed high-titer neutralizing antibodies during the first treatment cycle, thereby limiting subsequent administration such that only 21% of patients could receive a second round of treatment, which further compromised the anti-tumor efficacy of the drug.^95^

To overcome the limitations of SS1P in clinical applications, researchers developed a second-generation MSLN-targeting immunotoxin, LMB-100 (RG7787), based on SS1P. LMB-100, which consists of a humanized anti-MSLN fragment and a de-immunized PE24 (a modified fragment of Pseudomonas aeruginosa exotoxin), exhibited reduced immunogenicity and toxic side effects compared with SS1P in animal models.^119^ A Phase I trial evaluating the safety of LMB-100 in patients with mesothelioma or MSLN-positive solid tumors confirmed that the main adverse reactions were elevated serum creatinine levels and capillary leak syndrome, with most events being mild to moderate and within the clinically acceptable range. Regarding efficacy, of the ten mesothelioma patients who received either the 170 µg/kg or 140 µg/kg dose regimen, eight achieved stable disease and two experienced disease progression. Although LMB-100 exhibited lower immunogenicity relative to SS1P, most patients still developed neutralizing antibodies after two cycles of treatment, which limited retreatment capacity and thereby constrained the anti-tumor efficacy of LMB-100 as a single agent. Combination therapy strategies may represent a promising direction to optimize the efficacy of LMB-100.^96^

Preclinical studies have shown that combining paclitaxel with immunotoxins can enhance anti-tumor activity. However, in a clinical trial combining LMB-100 with paclitaxel in patients with advanced pancreatic cancer, this combination regimen resulted in severe adverse effects.^97^ Notably, subsequent studies revealed that some patients demonstrated expansion of CD4 + and CD8+ T-cell populations after receiving one cycle of LMB-100 treatment, suggesting that LMB-100 may elicit an immune response.^120^ In mesothelioma patients with disease progression after LMB-100 monotherapy, subsequent combination therapy with a PD-1 inhibitor achieved an objective response rate (ORR) of 40% and a median OS of 11.9 months among seven evaluable patients.^98^ Given the limited sample size in this trial, larger clinical studies are warranted to further verify the therapeutic value of this combination strategy.

#### 6.1.3 Antibody-drug conjugates

ADC are formed by covalently linking monoclonal antibodies to cytotoxic drugs (payloads) via chemical linkers. ADC specifically recognize antigens on the surface of tumor cells, and following cellular internalization, release their payload to selectively kill tumor cells.^121^ Many MSLN-targeted ADC have also been developed for cancer treatment, with their therapeutic effects demonstrated in preclinical studies. These agents not only effectively eliminate MSLN-expressing cancer cells, but also induce immunogenic pyroptosis, thereby enhancing immune responses.^122^

Anetumab Ravtansine (AR) is a novel ADC formed by coupling a humanized anti-MSLN antibody with the microtubule inhibitor DM4 via a disulfide-containing linker. In patient-derived xenograft tumor models, AR demonstrated high affinity for MSLN and was able to specifically recognize and kill MSLN-expressing tumor cells.^123^ In a Phase I trial of 148 patients with advanced solid tumors, there was one complete response (CR) and 11 partial responses (PR), resulting in an ORR of 8.1%. Additionally, SD was achieved in 66 patients. Common adverse reactions included fatigue, nausea, diarrhea, and vomiting. The MTD of AR was ultimately determined to be 6.5 mg/kg every three weeks.^99^ In another Phase II clinical trial targeting patients with MSLN-positive pleural mesothelioma, the median PFS was 4.3 months in the AR treatment group, compared with 4.5 months in the Vinorelbine treatment group. These comparable outcomes suggest that the efficacy of AR is similar to that of Vinorelbine.^100^

Platinum-based chemotherapy is an important component of standard chemotherapy regimens for various solid tumors, but platinum resistance often leads to treatment failure. To address this challenge, a study evaluated the efficacy of AR in combination with pegylated liposomal doxorubicin for the treatment of platinum-resistant ovarian cancer. Among the treated patients, 1 achieved CR and 17 achieved PR, yielding an ORR of 26.2%; the median duration of response (MDOR) and median PFS were 7.6 months and 5.0 months, respectively. Most adverse events associated with this combination therapy were Grade 1-2, and serious events were rare, confirming its feasibility.^101^ Another study investigated the combination of AR and pembrolizumab in the treatment of platinum-pretreated patients with pleural mesothelioma. The median PFS of the combination group was 12.2 months, which was numerically longer than that of the pembrolizumab monotherapy group (3.9 months), and no new dose-limiting toxicities were observed.^102^ These findings are of clinical interest; however, the trial had a limited sample size, and the therapeutic value of this combination strategy warrants validation in larger clinical trials.

BMS-986148 is a humanized antibody-drug conjugate targeting MSLN that is linked to the microtubule inhibitor tubulysin via a valine-citrulline linker. In the Phase I/IIa clinical trials for the treatment of advanced solid tumors, the safety profile of BMS-986148 was generally acceptable, regardless of whether it was administered as monotherapy or in combination with nivolumab. The most common adverse events included grade 1-2 hepatic enzyme elevations and fatigue. Preliminary efficacy analysis indicated an ORR of 6% for monotherapy, which increased to 20% in the combination therapy group, suggesting that BMS-986148 may synergize with nivolumab-mediated immune activation.^103^

Although traditional IgG1 antibodies (such as LMB-100 and AR) have demonstrated certain efficacy in clinical applications, their large molecular size often results in limited solid tumor penetration. To overcome this limitation, various miniaturized antibody fragments have been developed. Among them, the human heavy-chain variable (VH) single-domain antibody 3C9, owing to its small size, exhibits enhanced tissue penetration while maintaining high antigen-binding affinity. Additionally, as antigen-binding fragments derived from IgG antibodies, Fab fragments are widely used in immunotoxins and ADC due to their small size and favorable tumor penetration. However, their inherent structural instability and tendency to aggregate limit their broader clinical application.^124^ To address these stability issues, researchers have developed the novel FabCH3 antibody fragment, in which the CH1/CL domains of Fab are replaced with IgG1 CH3 domains. This modification not only significantly improves thermal stability and antigen affinity but also retains the penetration advantages conferred by its compact size.^125^ These miniaturized, high-stability antibody formats, including VH single-domain antibodies and FabCH3 fragments, effectively overcome the limitations of conventional antibodies in tumor penetration and stability. By markedly enhancing intratumoral distribution and therapeutic stability within the complex tumor microenvironment, these innovations represent significant progress in MSLN-targeted therapy and hold promise for facilitating the clinical translation of next-generation antibody therapeutics.

### 6.2 Cancer vaccine

Cancer vaccines are treatments that fight cancer by activating or enhancing the body's immune response. Technological advances have enabled significant breakthroughs in cancer vaccine development, opening new avenues for cancer treatment and prevention (Figure 5B).^126^

CRS-207, an engineered live-attenuated Listeria monocytogenes strain, can be phagocytosed by antigen-presenting cells as an intracellular pathogen. Once inside these cells, it drives robust expression of the MSLN-ActA fusion protein under the control of the ActA promoter. This MSLN-ActA fusion protein then enters the class I and class II antigen processing pathways, where MSLN is processed into antigenic peptides and presented on the cell surface. The presented MSLN antigenic peptides activate CD8⁺ and CD4⁺ T cells, thereby generating MSLN-specific T cells. Following a dose-escalation study, the maximum tolerated dose (MTD) of CRS-207 was defined as 1 × 10⁹ CFU in patients with advanced MSLN-positive cancers. MSLN-specific CD8+ T-cell responses were observed in 60% of patients treated at this dose, suggesting that the vaccine was effective in inducing anti-tumor immune responses. Additionally, 37% of patients had overall survival exceeding 15 months; however, with respect to tumor response, none achieved PR.^104^ In another clinical study of malignant pleural mesothelioma, CRS-207 in combination with chemotherapy achieved more favorable therapeutic outcomes. Of 35 evaluable patients, 86% achieved disease control. Specifically, 1 patient achieved CR and 19 achieved PR. Median PFS and OS were 7.5 months and 14.7 months, respectively. Immunological analysis showed that the regimen significantly enhanced intratumoral infiltration of T cells, dendritic cells, and NK cells, and the majority of treatment-related adverse events were of grade 1-2 severity.^105^

The GVAX vaccine elicits T cell immune responses against MSLN and may enhance therapeutic efficacy in combination with CRS-207. The feasibility of this therapeutic strategy has been evaluated in clinical trials. The triple combination of GVAX vaccine, CRS-207, and a PD-1 inhibitor yielded a disease control rate of 13.7% and a median overall survival of 5.9 months in patients with advanced pancreatic cancer, an efficacy comparable to second-line chemotherapy. By comparison, when GVAX vaccine was combined with cyclophosphamide alone, the objective response rate was only 2%-4%.^106^

Despite modest clinical outcomes from MSLN-targeted cancer vaccine trials, related basic research continues to advance. MSLN exhibits low immunogenicity, which poses a challenge for vaccine-induced immune activation. To address this issue, researchers are exploring ways to enhance the therapeutic efficacy of vaccines by enhancing the antigen-presenting capacity of dendritic cells. Among these, dendritic cell vaccines based on iPSC technology are an important research direction. Researchers have used genetic engineering techniques to fuse the MSLN gene with the ubiquitin gene, which enhances MSLN processing and presentation in dendritic cells, thereby increasing MSLN immunogenicity. Studies have shown that these genetically modified dendritic cells significantly enhance the anti-tumor activity of cytotoxic T lymphocytes.^127^ In addition, substantial progress has been made in combining MSLN vaccines with immune checkpoint inhibitors. MSLN-PD-L1 fusion vaccines exert synergistic antitumor effects by simultaneously eliciting MSLN-specific immune responses and blocking the PD-1/PD-L1 axis.^128^ These innovative strategies enhance dendritic cell-mediated antigen presentation and effectively counteract tumor immune evasion mechanisms, providing novel insights for clinical translational research on MSLN vaccines.

### 6.3 Oncolytic virus

Oncolytic viruses are an emerging strategy for cancer treatment. These genetically modified viruses can selectively infect and kill tumor cells.^129^ Beyond their direct cytotoxic effects, oncolytic viruses can stimulate antitumor immune responses and enhance the infiltration of immune effector cells, such as NK cells and cytotoxic T lymphocytes, to inhibit tumor growth and metastasis (Figure 5C).^130^

ONCOS-102 is a genetically engineered adenovirus that can target tumors and elicit antitumor immune responses. In a Phase I clinical study, ONCOS-102 demonstrated promising antitumor activity in patients with solid tumors. In patients with ovarian cancer, it induced a robust immune response against MSLN and enhanced the infiltration of CD8+ T cells.^107^ This study also showed that treatment with ONCOS-102 led to upregulation of PD-L1 in tumor cells, suggesting potential synergy with immune checkpoint inhibitors.

To enhance the safety of oncolytic viruses, researchers replaced glycoprotein D of herpesvirus with a high-affinity anti-MSLN antibody fragment, enabling the virus to selectively infect MSLN-positive cells, thereby minimizing damage to normal tissues. Additionally, the *IL-12* gene was inserted into the viral vector to enable infected tumor cells to continuously secrete IL-12, thereby activating immune effector cells in the tumor microenvironment and further augmenting the antitumor immune response. In a preclinical model, this oncolytic virus not only demonstrated potent oncolytic activity but also overcame the neutralizing effect of SMRP in the tumor microenvironment, ensuring sustained infectivity.^131^ These findings provide strong preclinical rationale for the application of oncolytic viruses in MSLN-positive tumors.

### 6.4 Chimeric antigen receptor T-Cell

Chimeric antigen receptor T-cell therapy (CAR-T therapy) is an innovative tumor immunotherapy approach. It involves genetically engineering a patient's own T cells to enable them to specifically recognize and eliminate tumor cells (Figure 5D).^132^ To date, MSLN-targeted CAR-T therapy has demonstrated promising antitumor activity and safety in preclinical models and early-phase clinical trials. With technological advances, this field has evolved from the traditional single-target strategy to novel designs such as dual-target CAR-T and logic-gated CAR-T, offering the potential for more precise and safer treatment options for malignant tumors.

While this therapy has shown remarkable efficacy in hematologic malignancies, its application in solid tumors still faces challenges.^133^ A Phase I study evaluating the safety and efficacy of MSLN-targeted CAR-T therapy demonstrated a favorable safety profile but limited antitumor activity. Among patients with malignant pleural mesothelioma, ovarian cancer, and pancreatic ductal adenocarcinoma, 11 patients achieved SD, with a median PFS of only 2.1 months. Tumor biopsies revealed insufficient CAR-T cell infiltration. Regarding safety, only one case of grade 4 sepsis was observed, with no off-target toxicity or cytokine release syndrome (CRS) reported.^108^

To further enhance the efficacy of CAR-T cells in solid tumors, the research team optimized the CAR construct by replacing the traditional CD3ζ chain with DAP12 and integrating the co-stimulatory molecule 4-1BB to synergistically promote T cell activation and proliferation. The newly constructed multi-chain DAP-CAR-T cells showed clear therapeutic potential in a single-arm, open-label, first-in-human clinical study. This study included eight patients with MSLN-positive advanced ovarian cancer or mesothelioma who had failed second-line or later treatments. The results showed an objective response rate of 33.3%, a disease control rate of 100%, and median progression-free survival and overall survival of 5.5 months and 10.5 months, respectively. In terms of safety, no severe neurotoxicity was observed, and the main adverse events were grade 1-3 cytokine release syndrome, all of which were effectively controlled with glucocorticoids or tocilizumab.^109^ Additionally, the expression of IL-7 and CCL19 has been proven to improve T cell infiltration and CAR-T cell survival in mouse tumors. Based on this mechanism, the anti-MSLN-7 × 19 CAR-T cells constructed showed superior migration ability to traditional anti-MSLN CAR-T cells in vitro studies and demonstrated stronger infiltration and killing effects on MSLN-positive pancreatic cancer cell lines (AsPC-1) in xenograft models. In a Phase I clinical trial, this therapy showed clear efficacy against MSLN-positive advanced solid tumors. One patient with advanced pancreatic cancer achieved CR 240 days after intravenous infusion, with significant shrinkage of local lymph node metastases and no new enlarged lymph nodes. Other patients achieved SD. In terms of safety, no grade 2-4 serious adverse events were observed. Only one patient experienced high fever, with no cytokine release syndrome (CRS) or neurotoxicity-related complications. Overall, the safety profile was manageable.^110^ Although both studies had the limitation of small sample sizes, they both confirmed that MSLN-targeted CAR-T therapy has clinical activity against advanced ovarian cancer, mesothelioma, pancreatic cancer, and other solid tumors that have failed multiple lines of treatment, providing a basis for further research.

Numerous studies have confirmed that targeting inhibitory immune checkpoints can effectively reverse CAR-T cell exhaustion^.^134^ To enhance the efficacy of CAR-T therapy, researchers have developed CAR-T cells capable of autocrine secretion of PD-1-blocking single-chain variable fragments (scFv). The scFvs secreted by these CAR-T cells are specifically enriched in the tumor microenvironment, effectively avoiding the off-target risks associated with systemic administration of PD-1 antibodies. In solid tumor models, these CAR-T cells release elevated levels of effector cytokines such as IFN-*γ* and IL-2. Additionally, the expression of CD80, CD107α, and Granzyme B in tumor-infiltrating lymphocytes is significantly upregulated, directly confirming enhanced T cell activity and cytotoxic function.^135^ Clinical trials evaluating this approach have been initiated. The combination therapy of αPD-1-mesoCAR-T cells (which autonomously secrete PD-1-blocking antibodies) and apatinib has demonstrated significant antitumor efficacy in patients with advanced ovarian cancer. Magnetic resonance imaging revealed a marked reduction in liver metastasis volume, with overall survival exceeding 17 months. The treatment was well-tolerated, with only mild adverse effects such as grade 1 hypertension and fatigue observed, and no severe immune-related adverse events such as cytokine release syndrome.^111^ This combination therapy shows promise, but larger-scale clinical trials are still needed to validate its long-term efficacy and safety.^

Tumor cells often exhibit heterogeneity in tumor antigens, which makes it difficult for single-target CAR-T cells to comprehensively recognize and effectively eliminate tumor cells.^136^ For example, by simultaneously targeting the FOLR1 and MSLN antigens, which are highly expressed in ovarian cancer, tumor cell elimination significantly increased from 48% with single-target therapy to 88.75%, effectively reducing the risk of recurrence caused by antigen loss.^137^ Exploiting the high but independent expression of MSLN and NKG2D ligands in triple-negative breast cancer, Saliu et al. designed a novel CAR-T cell that not only targets MSLN but also autonomously secretes bispecific T cell engagers (BiTE) targeting NKG2D ligands. These BiTE can recruit and activate bystander T cells in the tumor microenvironment to attack tumor cells that lack MSLN expression but express NKG2D ligands, thereby demonstrating superior tumor clearance and survival benefits in heterogeneous tumors.^138^ Collectively, these strategies indicate that overcoming antigen heterogeneity through multi-target approaches is a key direction for enhancing the efficacy of CAR-T therapy in solid tumors and demonstrate great potential for clinical translation.

### 6.5 Extracellular vesicles

Extracellular Vesicles (EV) have emerged as highly promising targeted carriers in the field of precision cancer therapy due to their inherent biocompatibility and intercellular delivery capacity. Researchers have successfully extracted functional exosomes from MSLN-targeted CAR-T cells. These exosomes fully inherit the CAR protein of CAR-T cells, enabling them to specifically target MSLN-positive tumor cells and directly induce tumor cell apoptosis using their carried cytotoxic granules (e.g., perforin and granzyme B).^139^

In addition to their direct cytotoxic effect, this type of MSLN-targeted exosome also serves as a multifunctional platform. Exosomes secreted by CAR-T cells can act as efficient carriers for delivering chemotherapeutic drugs such as paclitaxel. This not only increases the drug concentration at the tumor site but also significantly reduces systemic toxic side effects on normal tissues.^140^ Furthermore, besides CAR-T-derived exosomes, researchers have also successfully constructed engineered extracellular vesicle nanoparticles specifically targeting MSLN by modifying anti-MSLN antibodies on the surface of EVs through the biotin-streptavidin binding strategy. This platform can effectively load the TP53 tumor suppressor protein, precisely deliver it to TP53-deficient ovarian cancer, inhibit tumor cell proliferation and induce apoptosis by restoring TP53 expression, and successfully suppress SKOV-3 tumor growth in in vivo experiments.^141^ Compared with live cell therapy, this exosome/EV-based therapeutic strategy can effectively avoid severe toxic side effects such as cytokine release syndrome (CRS) and exhibits higher safety. However, even after MSLN-targeted modification, intravenously injected extracellular vesicles still tend to preferentially accumulate in reticuloendothelial system organs such as the liver and lungs, resulting in limited actual enrichment rate at the tumor site; meanwhile, the high osmotic pressure, acidic conditions, and matrix barrier of the tumor microenvironment will further hinder the penetration of vesicles into the deep tumor tissue, impairing their targeted killing effect.^142^ Therefore, tumor-targeted vesicles still need to optimize targeted delivery efficiency and administration methods (e.g., combining intravenous administration with intratumoral administration) to increase the local vesicle concentration at the tumor site and thereby optimize therapeutic outcomes.

## 7 The challenges and strategies of MSLN-targeted therapy

Under normal physiological conditions, MSLN is also expressed in the mesothelial cells of the pleura, peritoneum and pericardium. Therefore, MSLN-targeting therapeutic strategies must consider the potential risk of off-target toxicity.^143^^,^^144^ Clinical trials have shown that within 48 hours after CAR-T cell infusion, two subjects experienced Grade 3 adverse events, including tachypnea and hypoxemia, which eventually necessitated treatment discontinuation. The potential cause may be pulmonary inflammation or injury, which induces MSLN expression in benign lung epithelial cells. These MSLN-expressing cells are subsequently recognized by CAR-T cells, causing extensive lung injury.^145^ Therefore, in the clinical translation of MSLN-targeted therapeutic agents, it is essential to integrate them with validated companion diagnostics to accurately identify eligible patients and continuously monitor safety.^146^ Additionally, several strategies exist to reduce off-target toxicity. Nanoparticle encapsulation and controlled-release systems can optimize tissue distribution and sustained-release performance of therapeutics, thereby reducing the exposure of non-target organs to targeted drugs.^147^ Another strategy is to design CAR-T cells with conditional activation characteristics, in which CAR expression is regulated by specific signals in the tumor microenvironment. For example, CAR-T cells with IF-THEN logic gate circuits only express MSLN-CAR in the presence of tumor-associated fibroblasts.^148^ This condition-specific activation mechanism ensures that CAR-T cells are activated only in the tumor microenvironment, thereby preventing immune responses in normal tissues.

MSLN on the cell surface can be hydrolyzed by proteases to generate the soluble fragment SMRP.^7^ This may negatively impact MSLN-targeted therapy strategies. Once SMRP is released, it preferentially binds to therapeutic agents, thus preventing them from targeting tumor cells. Even when therapeutic agents successfully bind to MSLN on the cell surface, proteolysis may still lead to premature dissociation of the drug-MSLN complex, thereby compromising therapeutic efficacy. Researchers have conducted mass spectrometry analysis of patient ascites and cell supernatants. The results showed that the proteolytic cleavage sites of MSLN were predominantly located in the C-terminal region proximal to the membrane.^149^ Based on these findings, researchers developed the monoclonal antibody 15B6, which can bind to the membrane-proximal cleavage sites of MSLN and inhibit proteolytic cleavage. This approach reduces SMRP generation and enables antibody drugs or CAR-T cell therapy to more effectively target tumor cells.^150^ When designing MSLN-targeted therapeutics, targeting epitopes in the membrane-proximal region is an effective strategy to minimize SMRP interference and enhance therapeutic efficacy.

## 8 Perspectives

The study of tumor immune escape mechanisms has laid an important theoretical foundation for the development of novel immunotherapy strategies. Among these, MSLN acts as a crucial immunomodulatory molecule that shapes the immunosuppressive tumor microenvironment through various pathways. For instance, it can promote the polarization of M2-type tumor-associated macrophages by activating the CD24-Siglec-10 signaling axis; moreover, its high expression levels are negatively correlated with reduced CD8+ T cell infiltration. However, our understanding of the immunomodulatory mechanisms of MSLN remains limited. The impact of MSLN on key immune cell subsets such as neutrophils, NK cells, and antigen-presenting cells is not well defined, and its downstream signaling pathways and epigenetic regulatory networks in immunosuppression also need to be elucidated. Therefore, in-depth research into the regulation of various immune cells by MSLN and its interactions with other components in the tumor microenvironment will greatly deepen our understanding of tumor immune escape and open up new avenues for developing MSLN-based immunotherapies.

Beyond mechanistic studies, another key challenge for the clinical translation of MSLN is that its functions may vary across different tumor types. Although the oncogenic role of MSLN has been widely recognized, its specific roles and associations with prognosis differ among various cancers such as pancreatic cancer, ovarian cancer, and mesothelioma. Future research needs to conduct in-depth comparative analyzes of MSLN's signaling regulatory networks in different tumor microenvironments to clarify the specific conditions under which it exerts its oncogenic effects. These studies will provide a solid theoretical basis for personalized MSLN-targeted strategies based on different cancer types.

## 9 Conclusion

Targeted therapy represents a crucial direction for future cancer treatment, and the differential expression pattern of MSLN in tumor tissues offers a highly attractive opportunity for the targeted therapy of various malignant tumors. MSLN drives tumor progression by regulating malignant phenotypes such as tumor cell proliferation, invasion, and metastasis, and its expression level is also a potential prognostic biomarker for patients. Elucidating its molecular structural characteristics and biological functions will provide a solid theoretical foundation for the development of more selective targeted therapeutic strategies.

Currently, therapeutic strategies targeting MSLN have gradually expanded from traditional monoclonal antibody drugs to fields such as oncolytic viruses, cancer vaccines, and CAR-T cell therapy, with some clinical trials demonstrating certain antitumor efficacy. However, these strategies still face practical challenges including tumor heterogeneity, insufficient intratumoral drug penetration, and tumor microenvironment immunosuppression. Therefore, we believe future efforts should focus on combinatorial regimens integrating multi-target design and tumor microenvironment modulation. Single-targeting MSLN is prone to antigen escape and drug resistance due to tumor heterogeneity, while tumor microenvironment immunosuppression can further impair the efficacy of immunotherapies such as CAR-T. Multi-target designs can reduce tumor escape risk by targeting MSLN and co-expressed tumor antigens; combining strategies such as oncolytic viruses or cancer vaccines, or low-dose immune checkpoint inhibitors to modulate the tumor microenvironment, can further enhance therapeutic efficacy. Meanwhile, clinical translation needs to integrate biomarker detection for precise patient stratification, matching optimal regimens to individual patients and accelerating the clinical implementation of MSLN-targeted therapies.

## Data Availability

No data was used for the research described in the article.
